# Miso without kōji: nesashi miso ecology driven by spontaneous fermentation with *Mucor plumbeus*

**DOI:** 10.3389/fmicb.2026.1759987

**Published:** 2026-01-30

**Authors:** Caroline Isabel Kothe, Tiffany Mak, Achille Julienne, Kiyo Okazaki, Leonie J. Jahn, Joshua D. Evans

**Affiliations:** 1 Sustainable Food Innovation Group, The Novo Nordisk Foundation Center for Biosustainability, Technical University of Denmark, Kongens Lyngby, Denmark; 2 Microbial Foods Group, The Novo Nordisk Foundation Center for Biosustainability, Technical University of Denmark, Kongens Lyngby, Denmark; 3 Fermentation Cultures Group, The Novo Nordisk Foundation Center for Biosustainability, Technical University of Denmark, Kongens Lyngby, Denmark; 4 MATR Foods, Copenhagen, Denmark; 5 Department of Health and Nutrition, Shikoku University, Tokushima, Japan

**Keywords:** food fermentation, microbial ecology, metagenomics, genomics, pangenomics, spontaneous fermentation, traditional practice

## Abstract

Nesashi miso is a rare, traditionally fermented soybean paste from Japan, and unlike most misos is produced through spontaneous fermentation without the use of a kōji starter. Here we analyzed a nesashi miso alongside two other misos from the same producer (rice and black soybean) as well as a hatchō miso from another producer which, like the nesashi, is based only on soybeans. Shotgun metagenomics confirmed that while *Aspergillus oryzae* dominated the three kōji-based misos, nesashi miso lacked this starter culture, and revealed that it was instead dominated by other filamentous fungi, mainly *Mucor* spp. and *Penicillium* spp., and contained typical yeast and bacterial genera found in traditional misos such as *Zygosaccharomyces* and *Tetragenococcus*. Principal component analysis (PCA) of 65 publicly available metagenomes showed that the nesashi miso sample clustered with other spontaneous solid-state fermentations like Chinese qu rather than with traditional kōji-based misos. To further characterize this unique fermentation, we isolated the *Mucor* sp. from nesashi miso, and sequenced it using long-read genomic sequencing. Pangenomic analysis confirmed its identity as *M. plumbeus*, and revealed close relationships between food- and environment-derived strains, suggesting that some *Mucor* species may already be naturally equipped to grow, establish and function in food fermentation niches. The nesashi strain specifically shared a large core genome with *M. racemosus* C, a strain patented for use in food, suggesting the former’s potential for use in and potentially even adaptation to food environments. Functional annotation highlighted unique genes in the food strain group associated with amino acid metabolism, which may contribute to flavor formation. Together, these findings bridge traditional fermentation practices with meta/genomic insights, highlighting the built fermentation environment as a reservoir of potential starter cultures and the genus *Mucor* as a worthy candidate for future food fermentation research and innovation.

## 1 Introduction

Fermented foods have long been an integral part of the human diet, providing not only preservation but also nutritional, sensory, and functional properties (Marco et al., 2017; Tamang et al., 2020). Across cultures, fermentation practices have evolved through experimental knowledge and microbial domestication, giving rise to a remarkable diversity of food products—fermented dairy, meats, fish, vegetables, legumes and grains—each reflecting local environments and cultural traditions (Arrigan et al., 2024; Dunn et al., 2021; Gibbons and Rinker, 2015).

**Figure 1:**
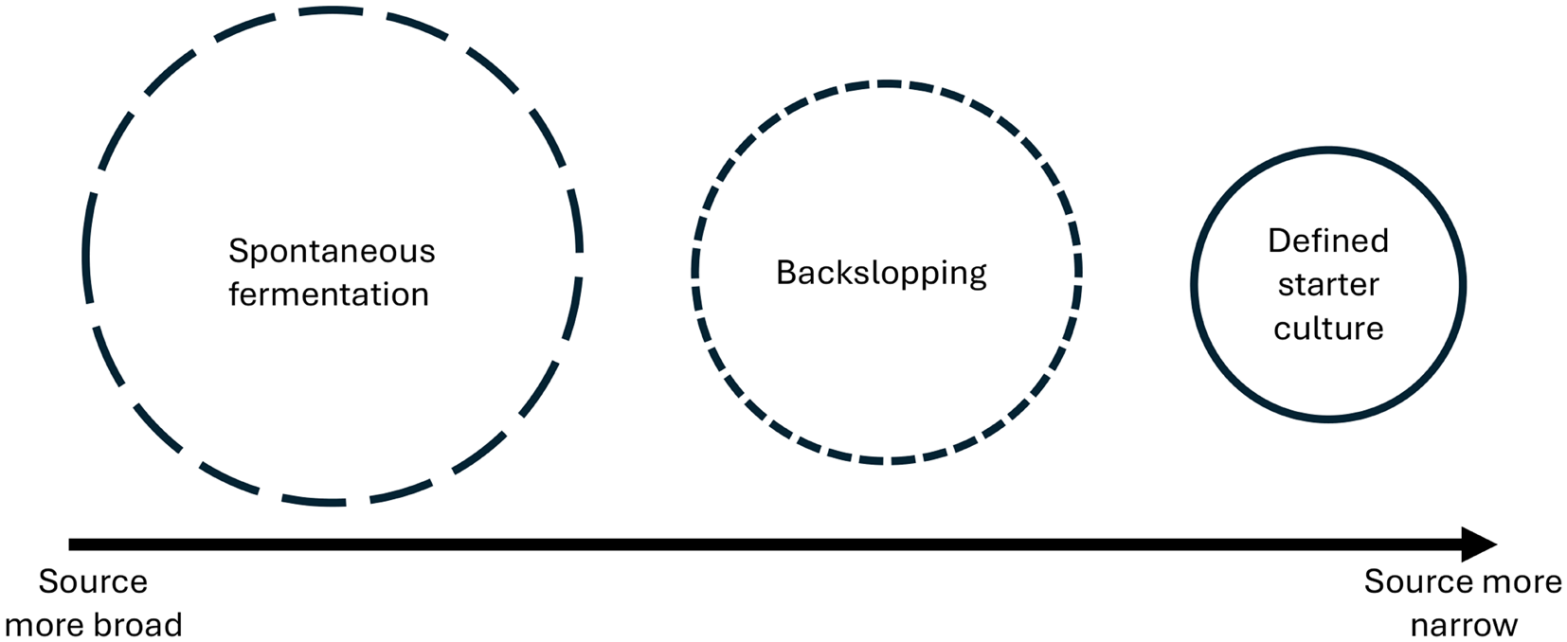
Continuum of modes of inoculation in fermented foods. The decrease in circle size represents the narrowing of the source of microbes, while the line porosity indicates consequent lesser openness to potential microbial diversity. This way of framing the relation between these common categories illustrates that microbial inoculation in food fermentations forms a continuum based not on degree of intention or control of the process (all can be more or less so), but on how defined the source of microbes is and how much additional diversity they may thus allow.

At the center of this diversity is the wide range of microbial inoculation strategies used in different fermented foods. In the literature, starter cultures are defined as microorganisms intentionally inoculated into food material to bring desired and predictable changes in the finished product (Durso and Hutkins, 2003). However, microbial inoculation in many traditional fermentations is far more complex. In cheese, for example, microbial communities may originate not only from added starters or ripening cultures, but also from the ‘house microbiota’ associated with humans, animals, milk, water- and airflows, brine, shelves, and processing equipment (Irlinger et al., 2015). Building on this broader perspective, we find it useful to consider fermentation systems as existing along a continuum of modes and sources of inoculation. Fermentations may be begun with pure or mixed defined starter cultures, but also through backslopping (microbes added from a finished batch of the same product), and/or through spontaneous fermentation, with microbes coming from the substrates and/or the processing environment (rooms, tools, equipment, and even people). Here we consider microbes added via known cultures, backslopping, and the environment as different kinds of inoculation (Figure 1). Doing so shows how they vary not by how intentional they are, as is often suggested, but by how defined the source of microbes is.

This continuum also helps to analyse more complex, multi-stage fermentations, such as miso. Miso is a traditional Japanese seasoning typically made with soybeans, salt, and kōji—usually *Aspergillus oryzae* grown on grain like rice or barley (Allwood et al., 2021; Kim et al., 2010; Kusumoto et al., 2021; Saeed et al., 2022).^1^ The kōji, most often made with pure culture inoculation, produces enzymes that help to break down the soybeans and turn the starches in the grain substrate into sugars, which then feed the microbial community in the miso, which spontaneously assembles, shaped by substrate, treatment, and environment (Kothe et al., 2024a,b). While most misos depend on kōji, a rare variety known as nesashi miso relies entirely on spontaneous fermentation.^2^ This traditional product ferments without any defined starter culture or even backslopping, depending on environmental inoculation and long aging to develop its characteristic flavor. Similar spontaneous processes are found in other Asian fermentations, such as Chinese douchi (fermented black soybeans), Korean doenjang (fermented soybean paste), and Indonesian ragi tapai (a spontaneously fermented starter culture based on rice flour), which rely on environmental microbes rather than defined starters (Nout and Aidoo, 2010; Xie and Gänzle, 2023). Studying nesashi miso therefore offers more than a description of a rare product; it provides another window into the biocultural diversity of Asian fermentations, highlighting the role of environmental microbes in ancient food traditions.

Scientific studies on nesashi miso are scarce. To our knowledge, only one study has been conducted, using culture-dependent and chemical methods, focusing on chemical changes during fermentation (Okazaki and Watanabe, 2023). This study showed how the miso’s long aging period drives biochemical changes, including Maillard reactions, darkening of color, and high accumulation of free amino acids compared with other miso types. Using culture-dependent methods, the authors identified the key fungus in the creation of the nesashi miso as *Mucor plumbeus*, which they detected only in the early stages of the miso fermentation, with no viable cells observed after 6 months of aging (Okazaki and Watanabe, 2023). Thus, the microbial ecosystem in nesashi miso and the functional contributions of *Mucor* in this fermentation remain poorly understood, which our study seeks to develop.

*Mucor* species are ubiquitous in natural environments, but representatives associated with food are scarce in genomic databases. Most publicly available *Mucor* genomes originate from environmental or clinical sources, reflecting their ecological versatility and their occasional pathogenic potential, as well as a likely sampling bias toward its more pathogenic contexts (Morin-Sardin et al., 2017; Walther et al., 2020). In food, *Mucor* can appear both through the addition of a specifically defined starter culture and as part of a spontaneously coalesced community. Its presence has been documented in surface-ripened cheeses (Marcellino and Benson, 2013; Metin, 2018), traditional African fermentations (Botha and Botes, 2014), and soybean-based fermentations, such as sufu (a soft, creamy, cheese-like fermented soybean curd) and douchi (Han et al., 2001; Xie and Gänzle, 2023). Some *Mucor* species can contribute to protein degradation and umami flavour development (Botha and Botes, 2014), suggesting that certain strains may have beneficial functional properties.

In this study, we employed culture-independent approaches to characterise the microbial composition of nesashi miso for the first time, and compared it with kōji-based misos produced using *A. oryzae*. We then integrated metagenomic datasets from misos and other spontaneous solid-state fermentations to contextualize its microbial profile with related products. Finally, we sequenced the first *Mucor* genome from a miso and performed comparative and pangenomic analyses with publicly available *Mucor* genomes from food and environmental origins, providing new insights into the genomic traits that may support the establishment of *Mucor* strains in fermented food ecosystems.

## 2 Materials and methods

### 2.1 Traditional production method of nesashi miso

**Figure 2:**
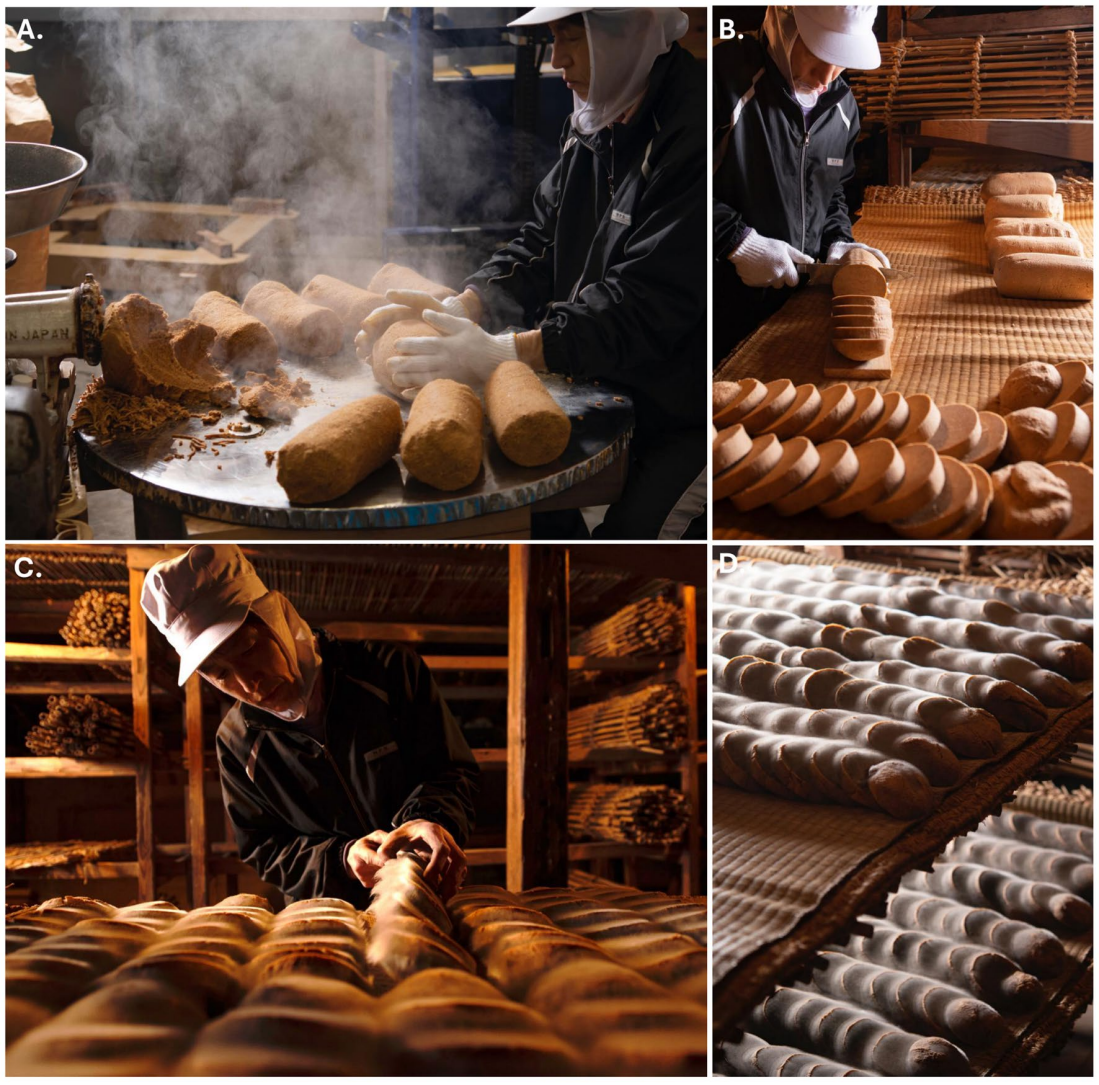
Traditional production of nesashi miso. Cooked soybeans are ground while still hot and shaped into cylindrical namako **(A)**, which are then sliced into ~2 cm pieces **(B)** and arranged on goza in the nesashi-doko for spontaneous fermentation in the kura **(C)**. After 15–20 days, the *Mucor plumbeus* that is dominant in the room begins to grow on the namako slices **(D)**. The artisanal producer shown in the images is Seiji Miura. *Image credit: Shogo Oizumi.*

The nesashi miso we study here is made by small-scale artisanal producers in Tokushima, Japan. The name ‘nesashi’ derives from the Tokushima dialect word *nesasu* (a dialectal form of the more standard *nekaseru*, 寝かせる), meaning ‘to let sit,’ referring to the long maturation time of the miso. For this reason, the term is sometimes used for long-aged miso in general; however, this nesashi miso is defined not only by age but by its traditional production method, including cold-season preparation (January-February) and particularly its spontaneous fermentation, a traditional method known as *shizen-bae* (自然生え, lit. ‘naturally occurring’). Soybeans are soaked and steamed in a *koshiki* (large wooden steamer) or boiled/steamed in a *hagama* (traditional cauldron). Once cooked, the liquid is drained from the soybeans, and is reserved, with salt added, and stored in the *kura*, or fermentary, used for fermentation since 1849. The beans, meanwhile, are put through a grinder while still hot. The hot soybean paste is then shaped by hand into cylindrical forms called *namako*^3^, and arranged on a *goza*—a thin mat woven from *igusa* (rush grass), to let them cool. The namako are then brought the next day to the kura. There, there is a *nesashi-doko*—a shelving area in a devoted room where the namako are left to rest. On top of the nesashi-doko, a *mushiro* (a mat woven from rice straw) is spread and a fresh goza is laid on top, where the namako, cut into 2-cm slices, are then arranged, and left to ferment spontaneously (Figure 2). This laying down of the namako slices invites the other meaning of ‘nesashi’, which is ‘to let sleep’. After 15–20 days^4^, a gray fungus that is dominant in the room (the *Mucor plumbeus*) begins to grow on the namako slices.^1^ After the namako becomes completely covered by the fungus, it dries out and becomes hard. It is then blended in a mixer with salt, water, and the reserved and salted soybean cooking liquid, and the mixture is transferred to cedar barrels and covered with a lid. The mixture is then left to mature for at least three years.^6^

### 2.2 Sampling and pH measurements

Four types of miso were analyzed in this study: (i) nesashi miso, a unique variety produced without kōji and spontaneously fermented; (ii) rice miso; (iii) black soybean miso (the three obtained from Miura Fermented Foods, 三浦醸造所, Japan); and (iv) hatchō miso (Maruya, まるや八丁味噌, Japan), a traditional Japanese organic miso produced exclusively from soybeans, included as a reference for comparison with nesashi miso, as both styles use only soybeans. Unlike the nesashi miso, the other three misos use kōji based on *Aspergillus* spp.

The pH of all miso samples was measured using a calibrated pH meter (Metrohm, model 913, Switzerland). Statistical analyses of the pH data were conducted using one-way analysis of variance (ANOVA), followed by Tukey’s *post hoc* test.

### 2.3 DNA extraction and sequencing

The DNA of the samples was extracted using the Qiagen DNeasy PowerSoil Kit, where 5 g of each miso were mixed with 45 mL of saline water (0.9% NaCl), placed in stomacher bags (BagPage, Interscience, France) and homogenized in a laboratory blender Stomacher 400 (Seward, Struers, Denmark) at high speed, for 2 min. This mixture was filtered (filter porosity of 280 microns) and subsequently the liquid was centrifuged to concentrate the cells. DNA samples were sent to BGI Group (Hong Kong, China) for metagenomic sequencing using the DNBseq PE150 platform.

### 2.4 Shotgun metagenomic analyses

Quality control and preprocessing of fastq files were performed with fastp v.0.23.2, using --cut_front --cut_tail --n_base_limit 0 --length_required 50 parameters (Chen et al., 2018). Taxonomic analysis of unassembled paired end reads was performed with Kaiju v1.9.0 (Menzel et al., 2016), using the NCBI BLAST nr+euk database. Taxa with relative abundances below 0.5% were excluded from downstream analyses. Taxonomic composition profiles were visualized in R v3.6.1 using the ggplot2 v3.3.2 package.

### 2.5 PCA with other miso and spontaneous solid-state fermented food metagenomes

To place nesashi miso microbiology in the context of other fermented foods, a comparative analysis was conducted using a total of 65 metagenomes: four from this study, 53 from miso studies (Coblentz et al., 2024; Kothe et al., 2024a,b), and 8 daqu samples (Yang et al., 2021).

Metagenomic reads were processed using the SIMKA tool (Benoit et al., 2016) to generate dissimilarity matrices based on the presence and absence of shared k-mers across samples. A k-mer size of 21 was applied (default and recommended parameter that offers a good balance between sensitivity and specificity for microbial metagenomes), and a presence-absence matrix was constructed using Bray–Curtis dissimilarity to assess microbial compositional differences.

Principal Component Analysis (PCA) was performed on the resulting dissimilarity matrix in R v.4.4.2, using the packages ggplot2 v.3.5.1, ggfortify v.0.4.17, and ggrepel v.0.9.5. PCA biplots were generated to visualize sample clustering.

### 2.6 *Mucor* sequencing and pangenomic analysis

*Mucor plumbeus* was previously isolated from nesashi miso using Potato Dextrose Agar (PDA) culture medium (Okazaki and Watanabe, 2023). For DNA extraction, fungal biomass and spores were suspended in Phosphate Buffered Saline (PBS) 1X, transferred into stomacher bags (BagPage, Interscience, France), and homogenized in a Stomacher 400 laboratory blender (Seward Co.) at high speed for 2 min. The homogenate was then transferred to 2 mL tubes and subjected to mechanical lysis using Precellys homogenization (FastPrep) for 2 × 30 s at 6,500 rpm, followed by vortexing for 15 min. The suspension was centrifuged to concentrate the cells, yielding a pellet of approximately 100 mg. DNA was subsequently extracted using the DNeasy PowerSoil Kit according to the manufacturer’s instructions.

Sequencing was performed using Oxford Nanopore technology. Libraries were prepared with the Native Barcoding Kit 96 V14 (Oxford Nanopore Technologies, UK), starting from 300 ng of genomic DNA, and loaded onto a MinION R10.4.1 flow cell. Basecalling was conducted using Dorado v.0.9.5 with the most accurate model and the --no-trim option. Read demultiplexing was performed with dorado demux, and read quality was evaluated using NanoPlot v.1.46.1. A total of 756,681 reads were generated, corresponding to 2,824,695,319 bases, with an N50 of 4,151 bp. The BAM files were converted to FASTQ format using Samtools v.1.21, and genome assembly was performed with Flye v.2.9.6 using the parameters --nano-hq --genome-size 50 m --asm-coverage 30. Reads were mapped to the assembly using Minimap2 v.2.25, and the genome was polished with Medaka v.2.0.1.

To assess the genomic relatedness between *Mucor plumbeus* from nesashi miso and other *Mucor* spp., all publicly available genomes of this genus were downloaded from NCBI,^7^ totaling 36 genomes. Genomes were then annotated with Augustus v.3.5.0, with the *Rhizopus oryzae* gene model selected as the closest available reference to *Mucor*, enabling the prediction of start and stop codons, introns, and exons. The predicted proteomes were then used for pangenomic analysis.

Core genome calculation was performed using Bacterial Pan Genome Analysis tool (BPGA) (Chaudhari et al., 2016), allowing the identification of conserved orthologs across the dataset. Conserved orthologs were extracted from each genome, and their amino acid sequences were aligned and concatenated to generate a super matrix for phylogenetic analysis. A phylogenetic tree was constructed with IQtree 2 (Minh et al., 2020), using the automated workflow available at ‘https://github.com/WeMakeMolecules/Core-to-Tree, with the *core_seq.txt* and *DATASET.xls* files generated as outputs from the BPGA run. Unique proteins clusters identified during pangenomic analysis were further annotated using the BlastKOALA server^8^ to assign functional categories.

### 2.7 Data availability

The raw sequences of genomic and metagenomic reads were deposited on the European Nucleotide Archive (ENA) under the BioProject ID PRJNA1348335.

## 3 Results

Our motivation was to determine how nesashi miso might differ from *Aspergillus*-kōji-based misos in microbial composition and functional genomic potential. To this end, we compared the four misos’ metagenomic profiles and pH values, positioned nesashi miso within a broader dataset of publicly available metagenomes from misos and spontaneous solid-state fermentations, and conducted pangenomic and functional analyses of a *Mucor* strain isolated from nesashi miso.

### 3.1 Nesashi miso differs from *Aspergillus*-kōji-based misos in microbial composition and pH

**Figure 3:**
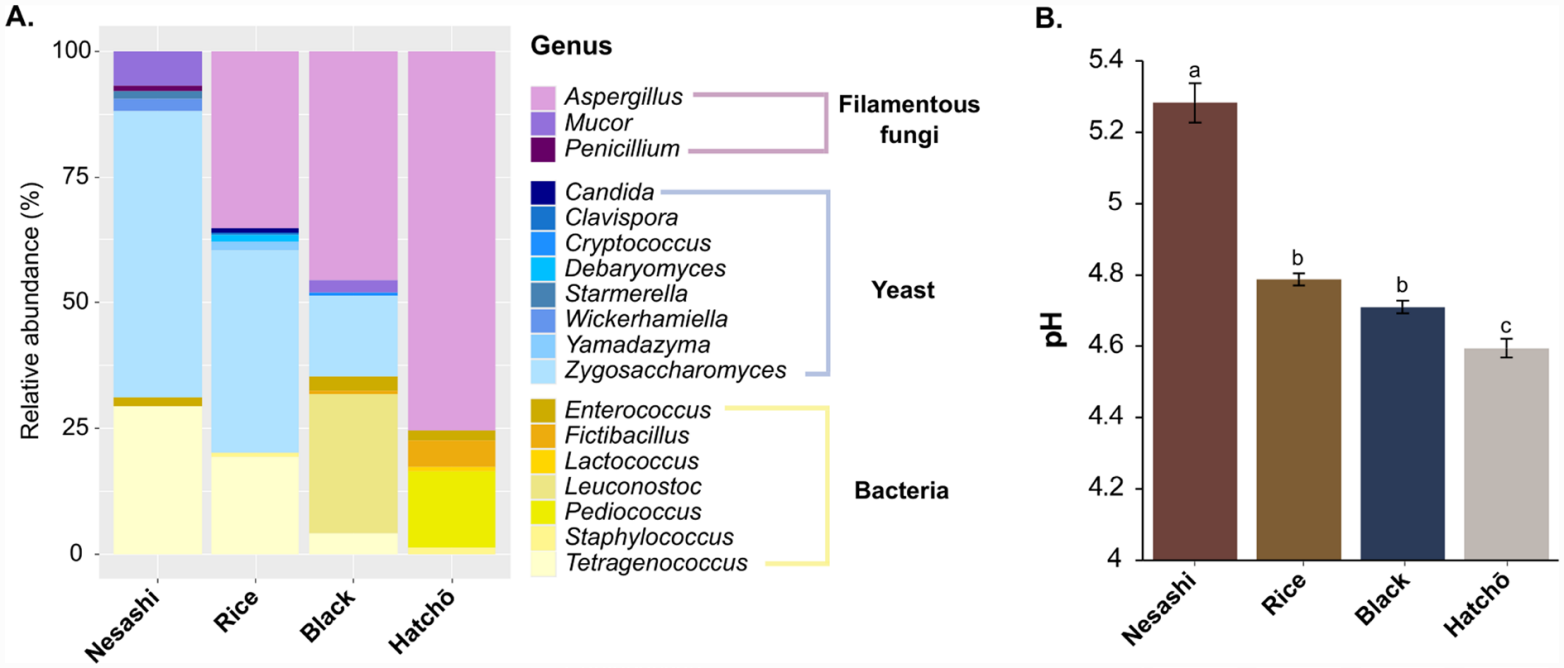
Microbial community composition and pH profiles across four miso types. Bar plots of the microbial composition of the misos at genus level **(A)** and pH variation across samples **(B)**. Letters in **(B)** represent significant differences.

Shotgun metagenomic analysis revealed that all analyzed samples contained filamentous fungi and bacteria (Figure 3A; Supplementary Table S1, ‘Kaiju’ sheet). Yeasts were also detected, except in the hatchō miso. *Aspergillus oryzae* was not detected in the nesashi miso, confirming its absence in this spontaneously fermented product. Instead, it was dominated by other filamentous fungi—mainly *Mucor*, along with *Penicillium*—as well as a high relative abundance of the yeast *Zygosaccharomyces* and the halophilic bacterium *Tetragenococcus*.

In the other misos, the dominant filamentous fungus was *A. oryzae*, confirming their use of kōji-based starter cultures. The main yeast in the rice and black soybean misos was *Zygossacharomyces*, while the main bacteria differed among these misos, with *Tetragenococcus* predominant in the rice miso, *Leuconostoc* in the black soybean miso, and *Pediococcus* in the hatchō miso.

Beyond these differences in microbial composition, nesashi sample exhibited the highest pH (5.2 ± 0.05), which was significantly higher than that of the rice, black soybean, and hatchō misos (Figure 3B; Supplementary Table S1, ‘Sample metadata’ sheet; ANOVA, *p* < 0.05, Tukey’s test).

### 3.2 Nesashi miso clusters with spontaneous solid-state fermentations rather than *Aspergillus*-kōji misos

The dataset compiled to microbiologically classify nesashi miso represents all shotgun metagenomic data for miso and spontaneous solid-state fermentations publicly available at the time of analysis (Supplementary Table S2). Only one study with publicly available metagenomic data on spontaneous solid-state fermentations was found, on daqu (Yang et al., 2021), highlighting the potential to further explore the microbiology of these diverse products.

**Figure 4:**
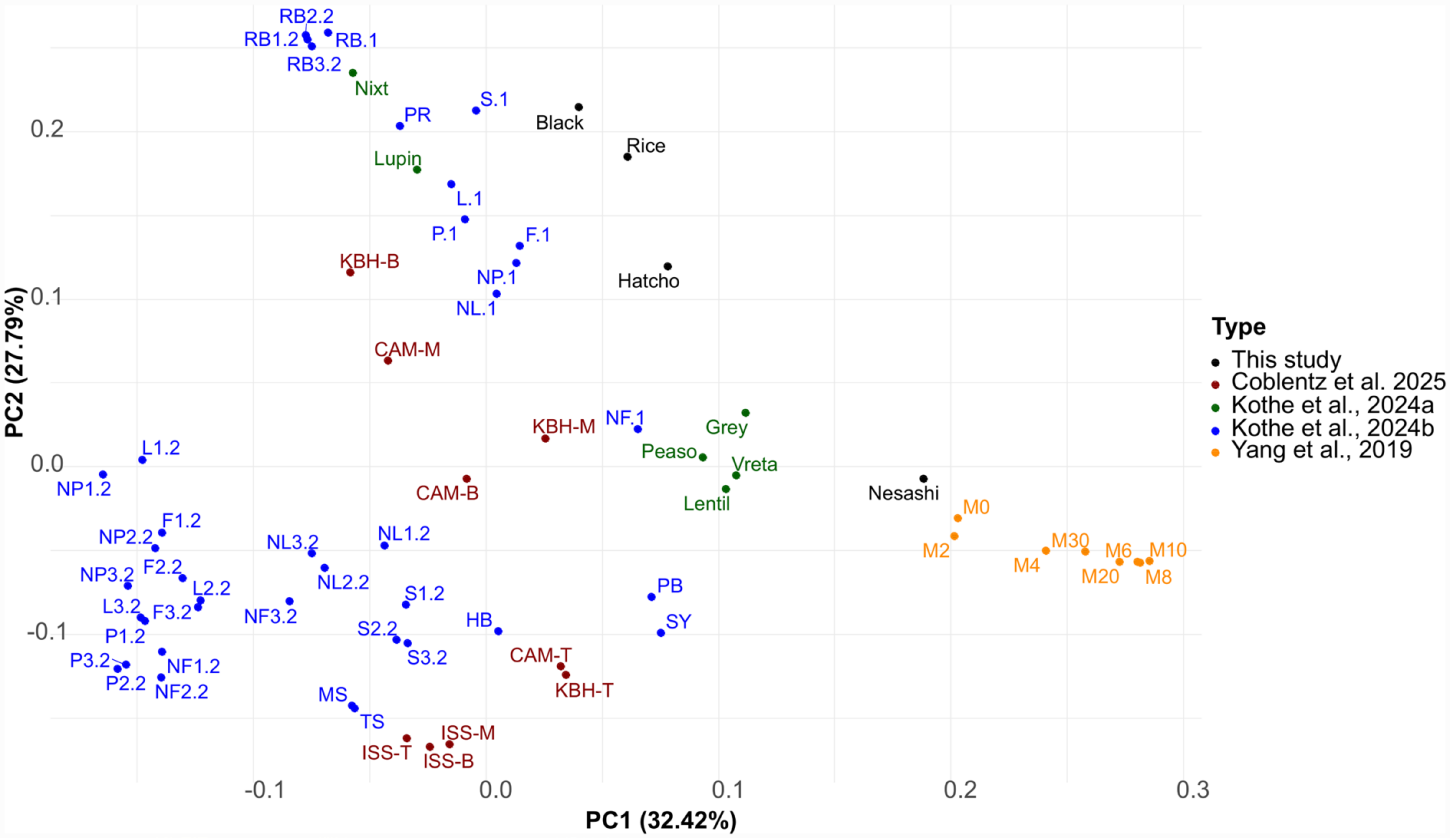
PCA of metagenomic profiles from 65 fermented food samples. The plot compares the spatial distribution of miso samples from this study (black points) with other miso types and solid-state spontaneous fermentations. The nesashi miso clusters more with the latter and not the former.

In the PCA, the nesashi miso clustered closely with other spontaneous fermentations, such as daqu, rather than with the kōji-based misos (Figure 4). The position of the nesashi sample in the ordination plot reflects its distinct microbial composition—previously shown to contain filamentous fungi such as *Mucor*, and lacking *A. oryzae* (Figure 3A).

### 3.3 Pangenomic analysis places the nesashi *Mucor* isolate among strains isolated from food and environmental sources

As *Mucor* was identified as the dominant filamentous fungus in nesashi miso, its genome was isolated and sequenced to confirm its taxonomic identity and determine its phylogenetic position relative to other *Mucor* strains. At the time of analysis, only 35 high-quality *Mucor* genomes were publicly available in the NCBI database (Supplementary Table S3, ‘Genome metrics’ sheet). Most originated from human sources, with a smaller number derived from environmental samples and only a few from food.

**Figure 5:**
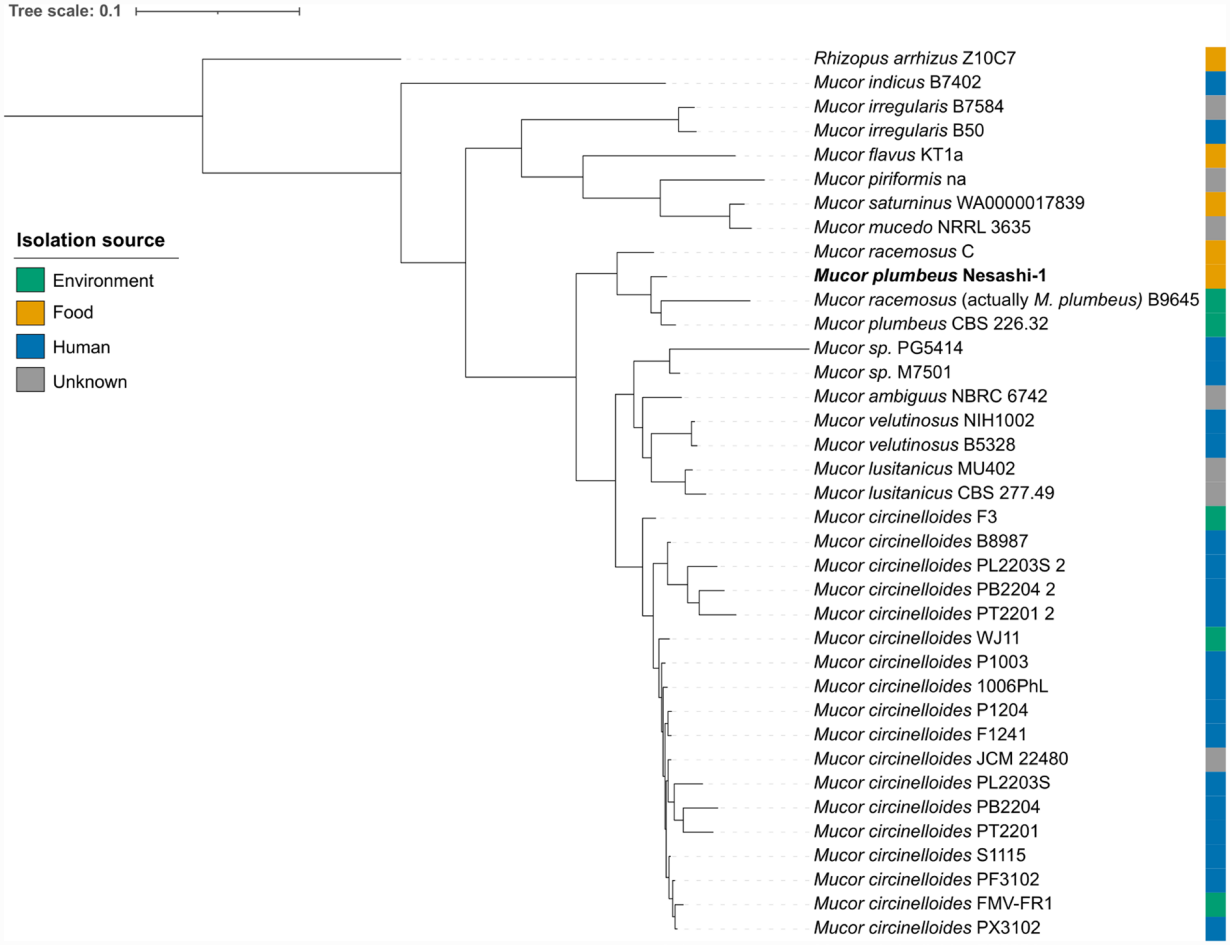
Pangenomic phylogenetic tree based on proteome sequences of *Mucor* genomes publicly available at the time of analysis. The tree includes the *Mucor plumbeus* strain from nesashi miso and other *Mucor* genomes isolated from food, environmental, and human sources.

Taxonomic assignment was performed using pangenomic clustering, followed by average nucleotide identity (ANI) against type and closely related reference genomes. Both analyses confirmed the nesashi isolate as *M. plumbeus*. The isolate clustered with two environmental *M. plumbeus* isolates (strain CBS 226.32, isolated from soil, and strain B9645, isolated from a clean-room floor) and one food-associated strain, *M. racemosus* C, previously isolated from douchi (Suo, 2024; Xie et al., 2023) (Figure 5). ANI analysis further indicated that the genome annotated as *M. racemosus* B9645 in the database actually corresponds to *M. plumbeus* (ANI > 99%), as we describe it above, suggesting a misclassification (Supplementary Table S3, ‘fastANI’ sheet). In this paper, we retained the publicly available species name for consistency with the database annotations, but suggest that it be understood as a strain of *M. plumbeus*, which we indicate in the Figure 5, Supplementary Table S3.

### 3.4 Functional genomic traits distinguish food-associated *Mucor* strains from environmental isolates

**Figure 6:**
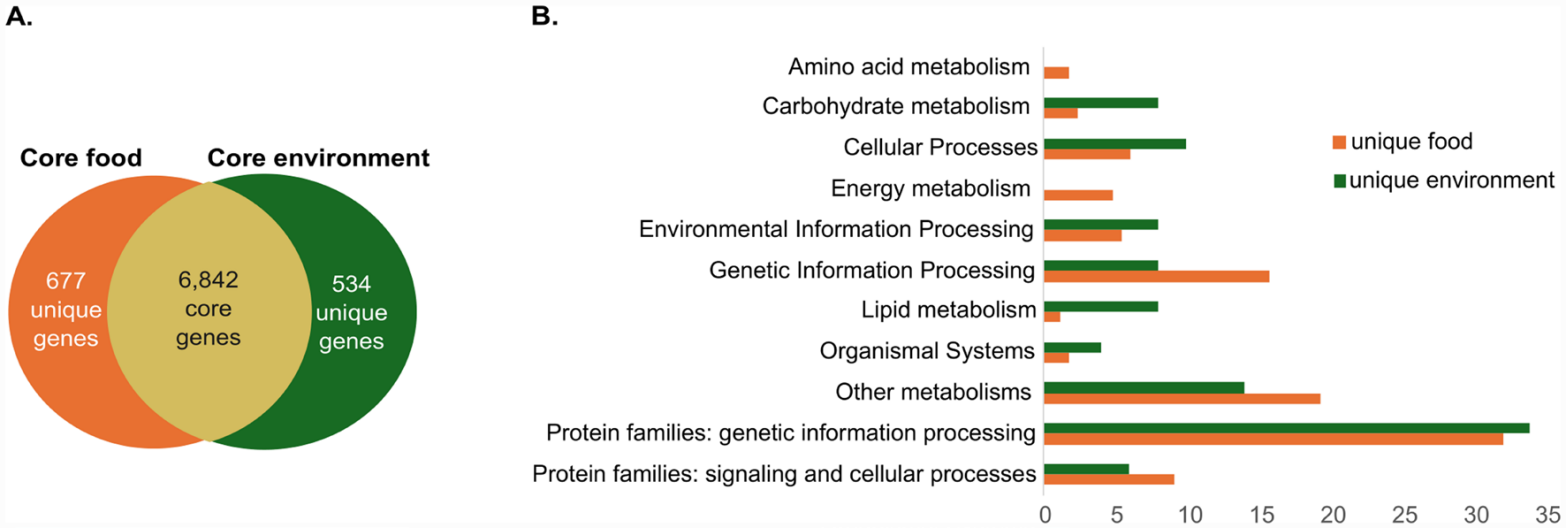
Comparison of unique gene functions between food- and environment-derived *Mucor* strains. Venn diagram of unique and core genes **(A)**. Proportion (%) of annotated genes across KEGG functional categories **(B)**.

To explore the potential functional distinctions between *Mucor* strains isolated from food and environmental sources, we focused on the *M. plumbeus*/*M. racemosus* cluster (Figure 5). The comparison of core genomes between the food-derived strains (*M. plumbeus* Nesashi-1 and *M. racemosus* C) and the environmental strains (*M. plumbeus* B9645 and CBS 226.32) revealed 6,842 shared genes, with 677 genes unique to food strains and 534 unique to environmental strains (Figure 6A). The proportion of annotated genes was higher among food-unique genes (24.4%) compared to environmental-unique ones (9.4%) (Supplementary Table S4). Within the annotated fraction, food strains harbored unique genes associated with amino acid metabolism, including enzymes linked to tyrosine (TAT, HPD) and histidine (CARNMT1) pathways, and energy metabolism mainly linked to oxidative phosphorylation (Figure 6B; Supplementary Table S4, ‘unique food’ sheet).

We further focused on the two food strains, which shared 7,727 genes. Our analysis revealed that 891 were unique to *M. plumbeus* Nesashi-1 and 571 unique to *M. racemosus* C. Only a small fraction of these unique genes could be annotated (7.6 and 6.7%, respectively). Despite belonging to different species, the two food strains shared a large number of core genes. Among the unique genes, the *M. plumbeus* Nesashi-1 contained genes associated with cell motility (Supplementary Table S4, ‘unique nesashi’ sheet).

## 4 Discussion

To place our findings in a broader context, we considered a larger research question: How does nesashi miso contribute to our understanding of the microbial ecology and functional potential of traditional spontaneous food fermentations?

### 4.1 Microbial signatures of nesashi miso

The only publicly available scientific literature on nesashi miso is an abstract presented at the Global Meet on Food Science and Nutrition Technology by Okazaki and Watanabe (2023). In their study, the authors described the traditional manufacturing process of nesashi miso and used culture-dependent methods to quantify *Mucor*, viable bacteria, and yeasts, without providing further taxonomic classification. As their observations were limited to culture-dependent techniques, the full microbial taxonomic diversity of nesashi miso has remained unexplored. This is why, in the present study, we characterized this artisanal product for the first time with a culture-independent approach, using shotgun metagenomics. We revealed a unique microbial profile dominated by filamentous fungi, mainly *Mucor*, as well as *Penicillium* (Figure 3A)—in contrast to the typical *Aspergillus oryzae*-driven fermentations of kōji-based misos. These fungi likely originate from the fermentation environment, as *Mucor* and *Penicillium* are common in spontaneous solid-state fermentations such as daqu and meju (Jung et al., 2014; Xiao et al., 2017; Zheng et al., 2011). While we detected *Mucor* in the finished miso and Okazaki and Watanabe (2023) found it only in the early stages of fermentation, we understand these results as compatible, as the *Mucor* itself became inactive in the miso environment after 6 months though its DNA remained. Beyond filamentous fungi, the ecology of nesashi miso also included halotolerant yeasts (*Zygosaccharomyce*s) and bacteria (*Tetragenococcu*s), genera typically found in misos (Allwood et al., 2021; Kothe et al., 2024a; Onda et al., 2003) and other salty fermentations, such as cheese rinds and soy sauces (Irlinger et al., 2015; Liu et al., 2023; Wen et al., 2024).

The presence of *Mucor* and *Penicillium*, and the absence of *A. oryzae*, may explain the higher pH observed in nesashi miso compared to the other misos analyzed in this study (Figure 3B). Traditional misos made with *A. oryzae* generally reach a wide range of final pH values, between 3.2 and 5.0 (Kothe et al., 2024a; Kusumoto et al., 2021; Onda et al., 2003). In contrast, nesashi miso exhibits a narrower and higher pH range of around 4.8–5.2, based on our measurements and the only available study to date (Okazaki and Watanabe, 2023). *A. oryzae* produces enzymes that break down starches and proteins during fermentation leading to the production of organic acids, such as L-malic and kojic acids, yielding this range of lower pH values (Kövilein et al., 2020; Yang et al., 2017). *Muco*r species, meanwhile, have been shown to metabolize organic acids and raise pH through aerobic metabolism, for example in yogurt acid whey (Fan et al., 2023). Similarly, in dairy cheese production, the curd is initially acidic, and the use of yeasts and filamentous fungi such as *Debaryomyces* and *Penicillium* during ripening contributes not only to flavor development but also to deacidification, through their utilization of lactic acid and/or breakdown of amino acids leading to the release of alkaline compounds such as ammonia (Fröhlich-Wyder et al., 2019; Spinnler, 2025).

Together, these metabolic and ecological patterns suggest that the dominance of *Mucor* may be not only a biochemical driver of deacidification but also a potential microbial marker of some spontaneous solid-state fermentations, particularly those based primarily on soybean and potentially those involving contact with rice straw and/or other plant materials. This could explain why nesashi miso clusters with Chinese daqu rather than with kōji-based misos in our PCA based on metagenomic data (Figure 4).

### 4.2 Is nesashi miso a miso?

The microbial distinctiveness of nesashi miso extends to its cultural identity. Its microbiological deviation from typical kōji-based misos raises a broader question: what defines a miso?

The typical understanding of miso prevalent in the scientific and technological literature is summarized by Allwood et al. (2021): “Miso production involves a two-stage fermentation, where first a mold, such as *Aspergillus oryzae*, is inoculated onto a substrate to make kōji. A subsequent fermentation by bacteria and yeast occurs when the kōji is added to a salt and soybean mash, with the miso left to ferment for up to 2 years.” This understanding is also common among practitioners of novel fermentations outside of Japan (Katz, 2012; Redzepi and Zilber, 2018; Shurtleff and Aoyagi, 1976). While there also exists much diversity even within this understanding—different substrates and ratios, different kōji strains and production methods, different ways to initiate the secondary fermentation (eg. spontaneously, through backslopping, or through inoculation of pure cultures), and different durations of fermentation—it captures the majority of miso production.

Yet there are other forms of miso traditionally made within Japan that do not fit this understanding, which are called and used as miso. ‘Misodama’, or miso balls, for example, are another traditional form of miso fermented entirely spontaneously, without the use of kōji inoculation. They are known to have been made, and to some extent still are, in more remote areas of the country, including Tōhoku (the northern part of Honshu, the main island), and central mountainous areas such as Nagano and Hyōgo prefectures. In this method, cooked soybeans are crushed, formed into balls, bound in rice straw, and hung to ferment and dry, sometimes above an oven so they also become smoked. Once fermented, they are made into miso by being mixed with salted water (Furukawa et al., 2013). This method closely resembles that of making meju in Korea, even more so than does nesashi miso.

It is likely that such practices of spontaneous solid-state fermentation are ancient, and were originally brought over to the Japanese islands from mainland East Asia, as part of the larger well-documented history of the introduction into Japan of various Chinese fermented products like jiangs. These became known as ‘hishio’ in Japan, which were the precursors for current-day miso (Shurtleff and Aoyagi, 1976).^9^ Thus these misos’ microbiological similarity to qu and their process similarity to meju is probably not a coincidence, but rather reflects their likely shared history. From this historically-informed perspective, though products like nesashi miso and misodama are now marginal relative to the most common ways of understanding and producing miso, they offer a reminder that the diversity of miso is even broader than is often thought, and invite us researchers to broaden our definitions to accommodate this existing diversity—in this case, to recognise that not all misos must be made with inoculated kōji.

This approach speaks to a broader question about how any fermented food is defined, by what disciplinary criteria, and by whom. As microbiologists, while it can be tempting to prioritise common production methods and their resulting typical microbiology in defining a given product, doing so can unduly narrow our understanding and appreciation of the full diversity of how the product is actually made in its cultural context. For this reason, we suggest that microbiological or technological definitions should always be placed in the historical and cultural context of the originating culture, in which the product was created, named, and transmitted. This approach aligns with recent calls to start from the originating culture’s understanding when conceptualising new forms of old foods (Samson et al., 2025), a logic which applies just as well to working with marginal cases of traditional foods. While nesashi miso diverges from more typical misos in its microbial ecology, its functional role in local food traditions, and the name given to it by its originating culture to signal this role, are what matter, and what make it a miso. This microbiological variation then becomes part of the diversity internal to miso as a category, rather than something that challenges its status as a miso. In general, whenever such divergence emerges with traditional products, the methodologically valid approach is thus to prioritise what came first, which is the product itself and its culture, rather than the scientific literature, and to then use the empirical case to update the conceptual definition in the literature, rather than to use the current conceptual definition in the literature to adjudicate the validity of the empirical case.

### 4.3 *Mucor* in food production

In fermentations like miso, the use of well-characterized and Generally Recognized as Safe (GRAS) species such as *A. oryzae* helps ensure consistent results as well as product safety (Daba et al., 2021). *Mucor*, despite its frequent presence in spontaneous fermentations, remains far less studied. Though some studies have explored its use as a starter in fermented foods, there is still limited knowledge on its role in food fermentation systems. This existing use, combined with the current lack of functional understanding, suggests that this genus holds underexplored yet potentially relevant functional potential.

Species of *Mucor* are widely distributed in nature, occurring in soil, air, and fermented food environments (Botha and Botes, 2014), which explains their frequent presence in spontaneous fermentations. Under certain conditions, however, some species can act as opportunistic pathogens or contaminants (Morin-Sardin et al., 2017; Walther et al., 2020), highlighting the need for accurate taxonomy and functional characterization when considering their application in food production systems.

Beyond their natural ubiquity, *Mucor* species have a long history of use in traditional fermented products across the world. In European surface-ripened cheeses such as Saint-Nectaire, Tomme de Savoie, and Swedish goat cheeses, *Mucor* contributes to rind development, proteolysis, and flavor formation (Båth et al., 2012; Marcellino and Benson, 2013). In China, fermented soybean products have traditionally relied on ‘house flora’ and more recently have been enabled by industrial pure cultures both of which often contain *Mucor* spp. For example, in *Mucor*-type douchi, *Mucor* qu is mixed directly with salts and seasonings, followed by the maturation fermentation stage (Xie and Gänzle, 2023). Another example is sufu (or furu), the cheese-like fermented soybean curd. Its production involves tofu preparation, pehtze^10^-making, salting, and ripening, during which *Mucor* can be one of the dominant microbes (Chen et al., 2022; Han et al., 2001; Li et al., 2021). Similarly, in Indonesia, ragi tapai, the spontaneous fermentation starter based on rice flour, also harbors *Mucor*, alongside other amylolytic microbes (Delva et al., 2022; Nout and Aidoo, 2010).

Across these diverse contexts, *Mucor* contributes its proteolytic and amylolytic activities, facilitating amino acid release and the formation of aromatic compounds such as umami and cheesy flavour notes (Tong et al., 2025; Yao et al., 2021; Zhang et al., 2023; Zhang et al., 2021). *Mucor* has also been explored for its technological potential in other fermentations, such as dry-fermented sausages (Selgas et al., 1995), highlighting its versatility in shaping flavor and texture across food products.

Our pangenomic analysis revealed that human-associated *Mucor* genomes clustered distinctly from those associated with food, suggesting niche-specific adaptation and possible functional specialization (Figure 5). However, this does not necessarily mean that those habitats are its unique niche. Unlike highly domesticated fungi such as *A. oryzae*, whose genomes show long-term human selection (Gibbons and Rinker, 2015; Machida et al., 2005), no comparable domestication signatures have yet been described for *Mucor* spp. Instead, available genomic data (Morin-Sardin et al., 2017)—including our results—support the hypothesis that *Mucor* maintains broad ecological flexibility and can transition between environmental and food contexts. For instance, the *M. plumbeus* isolate from nesashi miso showed close genomic relatedness to environmental *M. plumbeus* strains but also to *M. racemosus* C, the food-associated strain patented for use as a starter in food fermentation (Suo, 2024).^11^ This suggests that *Mucor* strains could be readily adaptable to a range of substrates, without requiring extensive genomic specialisation.

When comparing food-associated *Mucor* strains with strains of environmental origins, the food-associated genomes displayed unique genes related to amino acid metabolism. In particular, TAT (tyrosine aminotransferase) and HPD (4-hydroxyphenylpyruvate dioxygenase) are involved in the catabolism of tyrosine, producing intermediates that can lead to aromatic compounds (Parthasarathy et al., 2018; Shen et al., 2025), while CARNMT1 (carnosine N-methyltransferase), encoding a histidine methyltransferase, may contribute to histidine-derived methylation reactions with potential roles in nitrogen metabolism and flavor precursor formation (Shimazu et al., 2023). Amino acid catabolism during cheese ripening (especially from histidine, tyrosine, and methionine) has been linked to the formation of volatile and sulfur-containing compounds responsible for ‘umami’ and ‘cheesy’ flavor notes (Curtin and McSweeney, 2004; Le Quéré, 2011). This is consistent with recent findings showing that *Mucor* species can enhance texture and flavor complexity in plant-based cheese analogs (Chen et al., 2025). The presence of these genes in food-associated *Mucor* strains may support their involvement in flavor development through amino acid transformation.

Comparative genomics revealed close relationships between food- and environmental-derived strains, suggesting different and potentially complementary possibilities of ecological adaptation. First we observed that the *Mucor plumbeus* strain from nesashi miso possesses unique genes associated with cell motility, including for the synthesis of dynein and dynactin. We hypothesise that these genes could facilitate its adaptation to food matrices even when those may not be its typical habitat. This hypothesis is supported by evidence in several filamentous fungi species where genes related to endosome motility and the spatial organization of protein synthesis have been shown to play a role in hyphal growth (Steinberg, 2014), potentially facilitating dispersal and adaptation to different surfaces. Second, we observed that *M. racemosus* C shares a high number of core genes with the nesashi strain, despite belonging to a different species. The limited number of annotated differences between these strains suggests that environmental niches (the possible origin of the strain Nesashi-1) may already harbor strains pre-adapted to food-related habitats. Both mechanisms may have contributed to the adaptation of this *Mucor plumbeus* strain to the nesashi miso system.

Together, these findings reinforce the role of *Mucor* as an environmental fungus with relevant functional potential in food fermentation. The genomic overlap between food- and environment-derived isolates highlights the environment as a reservoir of microbes with desirable fermentation traits, offering opportunities for discovering new starter cultures.

## 5 Conclusion

In this study we developed a framework that relates the three common modes of inoculation in fermentation—spontaneous fermentation, backslopping, and defined starter cultures—based on how broadly defined their sources of microbes are. We then used this framework to analyze nesashi miso, a rare, traditional form of miso in Japan made without using kōji, where its inocula come solely from the environment. We revealed the composition of its microbial community and investigated the genomic functionality of its key fungal strain, *Mucor plumbeus* Nesashi-1—the first *Mucor* genome isolated from miso—and its relatedness to other *Mucor* strains. These results expand the limited scientific understanding of spontaneous miso fermentation. From them we argued for a broad approach to understanding miso that includes its diverse traditional forms, and suggested that *Mucor* likely has untapped potential for solid-state food fermentations, illustrating more generally how environmental fungi can adapt or may already possess traits that enable their establishment and functionality in food ecosystems.

Assessing the relative significance of these two hypotheses is a key direction for future work. Additional *Mucor* strains could be isolated from food and fermentation environments to expand the genomic and ecological comparisons presented here. Complementary assays—such as growth experiments and evolution tests conducted under food-like versus environmental conditions—could further clarify the ecological performance and adaptive and functional potential of these *Mucor* strains. These approaches could help determine whether the *Mucor* isolated from nesashi miso, as with other fungi isolated from spontaneously fermented foods, represents an environmental strain preadapted to thrive in food fermentation contexts, or a strain that has already begun adapting to this context—and more broadly suggest novel applications for *Mucor* spp.

Unlike many modern industrial fermentations, which often rely on defined starter cultures to enable predictability and control, artisanal production systems such as that for nesashi miso often rely on open, long-term interactions between substrates, tools, fermenters, and environment. Our findings align more broadly with a growing recognition that many key fermentative microbes originate from environmental sources such as plants, insects, and humans (Kothe et al., 2025; Sinotte et al., 2025). Nesashi miso thus exemplifies how ancient artisanal practices preserve microbial community assembly methods, in which the environment does not ‘contaminate’ food but acts as a cultivated microbial reservoir that shapes the fermentation process in desirable and predictable ways. Understanding such systems not only deepens our knowledge of microbial diversity in spontaneously fermented foods, but also suggests how these practices can be revalorized and further cultivated to reconnect modern food production with its environmental and cultural roots.

## Data Availability

The datasets presented in this study can be found in online repositories. The names of the repository/repositories and accession number(s) can be found in the article/Supplementary material.

## Footnotes

1 While kōji most often involves *Aspergillus oryzae* and sometimes other *Aspergilli* like *A. sojae*, *A. luchuensis*, or *A. awamori*, in Japan it can also refer to a much broader set of genera including *Rhizopus* spp., *Monascus* spp., Mucor spp., and *Absidia* spp. when grown on grains or legumes (Shurtleff and Aoyagi, 1976).

2 To give a sense of the relative rarity of nesashi miso: the total domestic production of all types of miso in Japan in 2024 was 456,487 tons (MAFF, 2024), and the annual production of nesashi miso is typically around 0.5 tons per year.

3 The Japanese word for sea cucumber.

4 In the past, this phase lasted 40–60 days. However, recent winter warming has altered the process: longer rest periods now favor blue mold and other undesirable molds. Producers monitor temperature and humidity and typically allow at least 15 days, extending the period when conditions permit.

6 While the exact maturation period for nesashi miso is not dictated by law, a long maturation is typical. Another published description of nesashi miso notes that it develops its characteristic flavor and aroma after more than 2 years of aging (Miso Health Promotion Committee, 2001, pp. 345–7). The exact duration varies by producer.

7
https://www.ncbi.nlm.nih.gov/datasets/genome/?taxon=4830

8
https://www.kegg.jp/blastkoala/

9 Not much is known about when and how miso began to use kōji instead of spontaneous fermentation, but it seems to have been a gradual process rather than a single event. Making kõji based on pure strains of *A. oryzae* has been practiced in Japan since at least the Edo period (from 1603 CE), if not earlier (Lee, 2018, 2021; Yamashita, 2021).

10 Tofu cubes inoculated spontaneously with the ‘house flora’ or with defined fungal cultures.

11 The inventor says that this starter culture is “suitable for the fermentation of fermented black beans, fermented bean curd, bean curd or soy sauce, and provides a unique flavor of *Mucor*-type fermented black beans.”
