# Supplementary material for: Miso without kōji: nesashi miso ecology driven by spontaneous fermentation with Mucor plumbeus
Source: Front Microbiol. 2026 Jan 30;17:1759987. doi: 10.3389/fmicb.2026.1759987 (PMC12901402; doi:10.3389/fmicb.2026.1759987)
Supplement: Supplementary file 1 [file Data_Sheet_1.pdf]

Kothe et al. 2026 'Miso without kōji'

Supplementary Material

Supplementary Tables

Table S1. Metadata and taxonomic profiling of samples analysed in this study.

Table S2. Dataset used for principal component analysis (PCA) of metagenomes.

Table S3. Quality metrics of *Mucor* genomes used for pangenomics, and pairwise Average Nucleotide Identity (ANI) used to assess genomic relatedness between *Mucor* isolate and reference genomes.

Table S4. Comparison of unique gene functions between food- and environment-derived *Mucor* strains.

Supplementary Author Contribution Information

JE conceived of the study and assembled the collaboration team. KO shared her *Mucor* isolate from the nesashi miso and further background information on its production. CK, TM, and JE conducted preliminary metagenomic analyses on the nesashi miso ecology and PCA comparison. AJ sequenced the genome of the *Mucor* isolate. CK conducted further pangenomic and comparative analyses on the *Mucor*, with feedback from TM, AJ, LJ, and JE. JE provided funding for the analyses. CK wrote the first draft of the manuscript, which she and JE revised with input from the other authors. All authors read, reviewed, and approved the manuscript before submission.
